# Effective Solvent‐Engineered All‐Inorganic CsPbI_2_Br Perovskite Solar Cells

**DOI:** 10.1002/advs.77395

**Published:** 2026-08-27

**Authors:** Rajarshi Roy, Mahdi Malekshahi Byranvand, Tim Kodalle, Maximilian Krappel, Laxman Gouda, Stephan Boehringer, Chittaranjan Das, Mayank Kedia, Carolin M. Sutter‐Fella, Michael Saliba

**Affiliations:** ^1^ Institute for Photovoltaics (ipv) University of Stuttgart Stuttgart Germany; ^2^ IMD‐3 Photovoltaics Forschungszentrum Jülich Jülich Germany; ^3^ Molecular Foundry Lawrence Berkeley National Laboratory Berkeley California USA; ^4^ Advanced Light Source Lawrence Berkeley National Laboratory Berkeley California USA; ^5^ Institute of Physical Chemistry University of Stuttgart Stuttgart Germany; ^6^ CeNSE Department Indian Institute of Science Bangalore India

**Keywords:** chemical engineering, crystallization, fourier transform infrared spectroscopy, materials science, perovskite, solvation shell, solvation, solvent, surface roughness, triple junction

## Abstract

All‐inorganic CsPbI_2_Br perovskite composition has attracted considerable attention in recent years due to its thermal robustness, convenience for space applications as well as for all‐perovskite triple junction applications as a top cell due to its wide‐bandgap (1.9 eV) nature. However, homogeneous film formation, perovskite α‐phase stability, poor crystallization, and solvent trapping still remain as the major challenges for this composition. Here we report a detailed analysis of solvent engineering strategy upon addition of AcN to DMSO to weaken the DMSO‐rich solvation shell as supported by DLS, FTIR, GIWAXS and device data and facilitate the residual solvent removal followed by controlled crystallization resulting in better film morphology, lower surface roughness, reduced trap‐assisted recombination and improvement in device parameters from 12.7% (untreated) to 14.0% (AcN‐treated).

## Graphical abstract

All‐inorganic CsPbI_2_Br perovskite composition has gathered a lot of attention in recent years due to its thermal robustness, convenience for space applications as well as for the all‐perovskite triple junction applications as a top cell due to its wide‐bandgap (1.9 eV) nature. However, homogeneous film formation, perovskite α‐phase stability, poor crystallization, and solvent trapping still remain as the major challenges for this composition. Here we report a detailed analysis of solvent engineering strategy upon addition of acetonitrile (AcN) to dimethyl sulfoxide (DMSO) to weaken the DMSO‐rich solvation shell as supported by dynamic light scattering (DLS), Fourier‐transform infrared spectroscopy (FTIR), grazing‐incidence wide‐angle X‐ray scattering (GIWAXS) and device data and facilitate the residual solvent removal followed by controlled crystallization resulting in better film morphology, lower surface roughness, reduced trap assisted recombination and improvement in device parameters from 12% (Untreated) to 14% (AcN‐treated).

## 1 Introduction

In recent years, metal halide perovskite solar cells (PSCs) have shown major improvements in power conversion efficiencies (PCE), now standing at a record ∼27% [1]. These record values, however, are achieved in hybrid perovskites with organic cations such as methylammonium or formamidinium. This imposes long‐term stability concerns against, e.g., heat [2]. Thus, in recent years, all‐inorganic compositions have attracted considerable interest using, e.g., inorganic cesium ions (Cs^+^) as cations. By varying the halide composition, CsPbX_3_ (with X being I or Br) spans a tunable bandgap range from 1.7 eV (for CsPbI_3_) to 2.29 eV (CsPbBr_3_) [3, 4, 5, 6, 7]. Unfortunately, CsPbI_3_ exhibits phase instability at room temperature. Starting with CsPbI_2_Br (at 1.89 eV), a phase‐stable material at room temperature emerges, which is more suited for single‐junction solar cells compared to CsPbBr_3_ (2.29 eV). The tuning of the halide ratio also improves the crystallization of the perovskite thin films. In addition, solvent engineering also facilitates improved nucleation and crystallization [8, 9]. Generally, the all‐inorganic perovskite precursors are well dissolved in pure dimethyl sulfoxide (DMSO) due to its high dielectric constant and polarity [10]. As the old chemistry adage goes, “like dissolves like”; when perovskite precursors (CsI, PbI_2_, PbBr_2_) are mixed with DMSO, the Pb^2+^ coordinates with the electron lone pairs of sulfur (S) from DMSO and forms a coordination compound, i.e., the [DMSO‐PbI_2_] adduct [11]. This adduct complex formation further helps to stabilize the perovskite intermediate phase toward more controlled crystallization [12, 13, 14]. However, one major hurdle is the usage of the slowly degassing DMSO, which is challenging to remove during the film annealing. This results in trapped solvent, leading to poor nucleation and film morphology. To avoid this issue, many strategies have been implemented over time, including tuning the annealing temperature and different fabrication methods [15] such as the spin‐force method [16] or mixed solvent engineering [17, 18, 19]. Especially, solvent engineering plays a direct role in controlling the perovskite film crystallization as well as reducing trap states in the perovskite crystal lattice. Here, we study solvent engineering of the perovskite precursor by partially substituting DMSO with acetonitrile (AcN), obtaining a perovskite layer with less trapped solvent, which leads to improved thin films.

AcN is a less polar solvent with a dielectric constant of 35.09 compared to DMSO, which has a higher polarity with a dielectric constant of 46.83. AcN does not dissolve the perovskite precursor materials fully, but as a co‐solvent of DMSO with an appropriate ratio dissolves the perovskite precursors fully. On the other hand, AcN has a low boiling point of 82°C compared to DMSO at 189°C. Importantly, the mixture of these two solvents exhibits a different overall boiling point compared to pure DMSO due to the formation of an azeotrope. However, for the [DMSO‐AcN] system, the solvent evaporation behavior cannot be obtained solely by the boiling points of the pure solvents since the perovskite precursor salts alter the solvent activity followed by evaporation dynamics (in this case, [DMSO‐AcN], [DMSO‐Pb] interactions). This phenomenon can also influence the evaporation dynamics, leading to relatively faster solvent extraction during perovskite annealing and resulting in improved film morphology. As a result, our solvent engineering strategy boosts the PCE from 12.7% to 14.0%.

## 2 Results and Discussion

**FIGURE 1:**
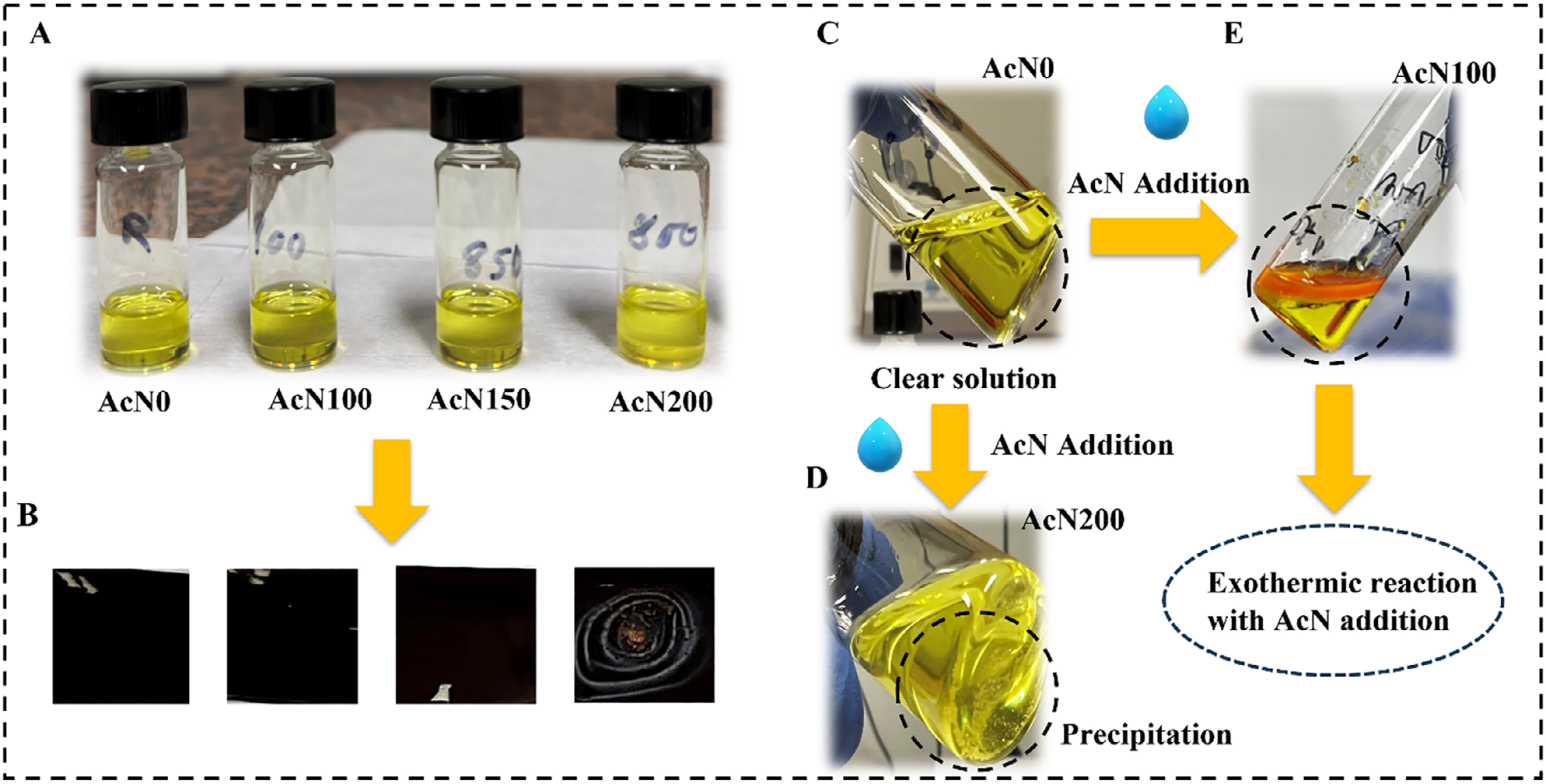
(A, B) Represent perovskite solutions and the corresponding perovskite films with varying ratios (v:v) of DMSO and AcN; (C) represents the clear perovskite solution obtained with AcN100, (D) represents the precipitation of the perovskite salts in AcN200, (E) represents the exothermic reaction occurring during the addition of AcN in DMSO‐based perovskite solution.

CsPbI_2_Br perovskite precursors with different ratios of AcN:DMSO (v:v) were studied, starting from 100:900 to 150:850 to 200:800. For convenience these will be referred to from here on as AcN0 (no AcN is added to DMSO), AcN100, AcN150 and AcN200 as can be seen in Figure 1. We observed that adding AcN to DMSO results in an orange‐colored phase appears temporarily for the AcN100. We hypothesize further below that this could stem from the formation of a plausible [DMSO‐AcN] adduct. After AcN150, the perovskite precursors are no longer fully dissolved as AcN is added to the solution, resulting in poor morphology and inhomogeneous perovskite films, see Figure 1. We attribute it to the less polar nature of AcN relative to DMSO as well as to the electron‐accepting nature of AcN.

**FIGURE 2:**
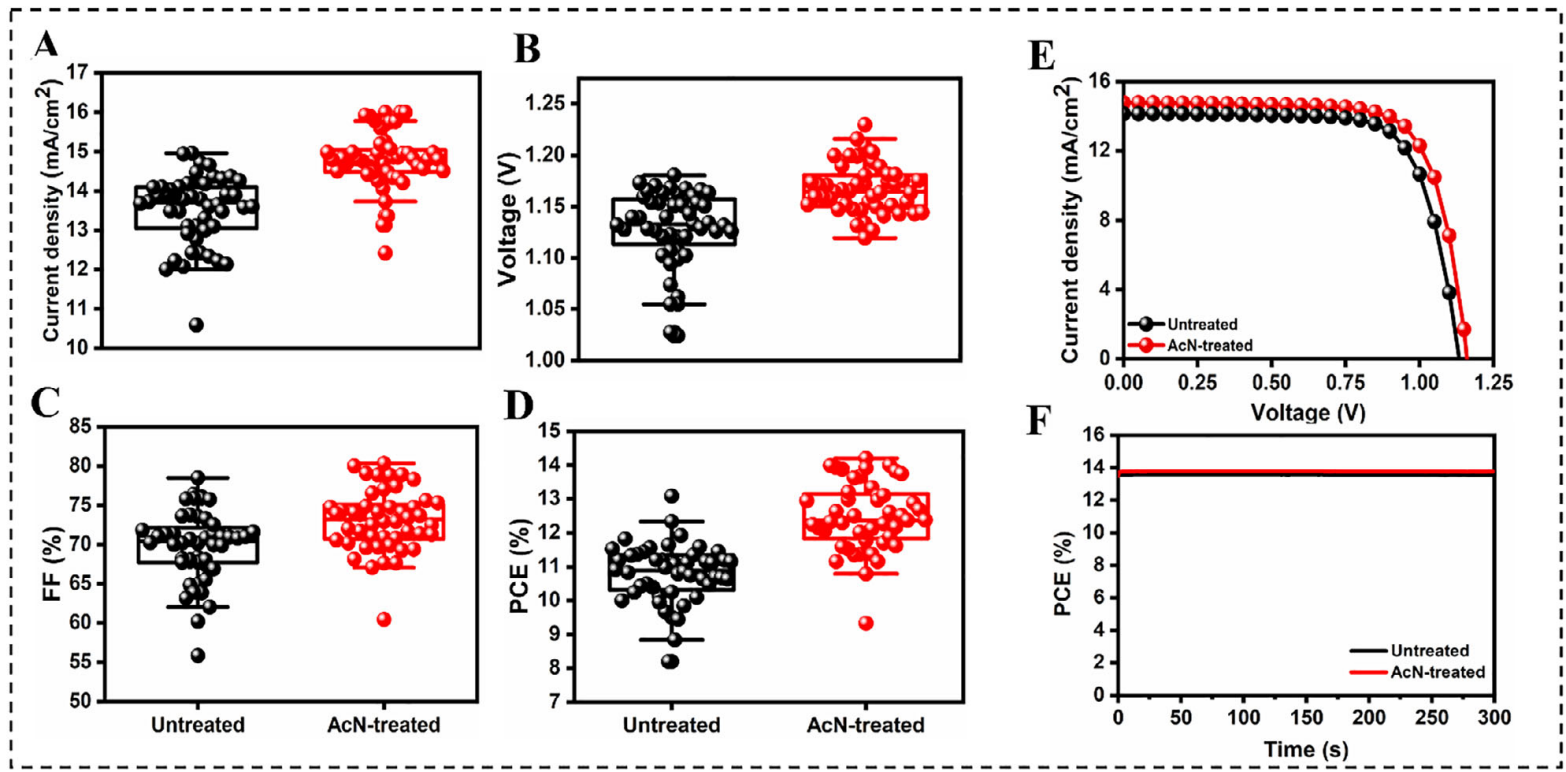
(A–D) represent device statistics (*n* = 48) of the untreated and AcN‐treated devices for *J_SC_*, *V_OC_*, *FF*, and PCE. (E) *J*–*V* measurement and (F) stabilized power output (SPO) at 0.95 V for the champion untreated and AcN‐treated devices.

Based on the different solvent ratios, different CsPbI_2_Br perovskite films are deposited for fabricating the PSCs with planar configuration, consisting of indium tin oxide on glass/nanoparticle‐based SnO_2_ electron transport layer/CsPbI_2_Br light absorber layer/poly(3‐hexylthiophene‐2,5‐diyl) hole transport layer/gold metal electrode. Maintaining the same variations for the AcN: DMSO, perovskite precursors were dissolved, followed by device fabrication as shown in Figure 1. The best device performance was obtained for AcN100. Since AcN has a lower boiling point of 82°C compared to DMSO at 189°C, the AcN was added to the perovskite precursor solution before deposition. For convenience, from here, the perovskite devices and films with this AcN:DMSO optimum ratio are referred to as “AcN‐treated” (i.e., AcN100) compared to AcN0 as “untreated”. During the device fabrication, the state‐of‐the‐art two‐step annealing process has been used, i.e., at 50°C followed by 160°C annealing. The 50°C step initiates the crystallization; the 160°C step predominates the residual solvent removal from the system. The untreated PSCs show an average PCE of 11.0 ± 1.7%, open‐circuit voltage (*V_OC_*) = 1.12 ± 0.04 V, short‐circuit current density (*J_SC_*) = 13.0 ± 1.0 mA/cm^2^ and fill factor (*FF)* = 73 ± 2.0% and the AcN‐treated devices show an average PCE of 12.0 ± 2.1%, *V_OC_* = 1.15 ± 0.04 V, *J_SC_* = 14.0 ± 1.0 mA/cm^2^ and *FF* = 76 ± 2.0% as shown in Figure 2A–D. Figure 2E shows the champion current density–voltage (*J*–*V*) curves for the untreated and AcN‐treated devices. The *V_OC_* improved from 1.14 to 1.19 V, the *J_SC_* improved from 14.8 to 15.0 mA/cm^2^, the FF improved from 74% to 78%, leading to the overall PCE from 12.7% to 14.0% for untreated and AcN‐treated, respectively. Figure 2F represents the stabilized power output under constant illumination for 300 s, showing impressive stability for the AcN‐treated device. Device statistics with varied AcN:DMSO (v:v) solution mixture can be found in Figure S1 together with the respective hysteresis index (HI) in Figure S2, where the HI decreases from 1.8% to 0.8% for the untreated and AcN‐treated devices, respectively, with the scan rate of 50 mV/s. This HI reduction is also supported by fewer trap‐assisted recombinations by the addition of AcN, which will be discussed in the time‐resolved photoluminescence (TRPL) data discussion, where the untreated film goes through faster decay, whereas the AcN‐treated film shows relatively higher long‐term stability. Furthermore, we have carried out robust thermal stability tests for both untreated and AcN‐treated devices at 200°C for 240 min and humidity tests at approximately 60% relative humidity for 180 min as shown in Figures S3–S6, respectively. In both conditions, the AcN‐treated devices outperformed untreated ones, proving the efficacy of AcN‐related co‐solvent engineering for all‐inorganic perovskite.

**FIGURE 3:**
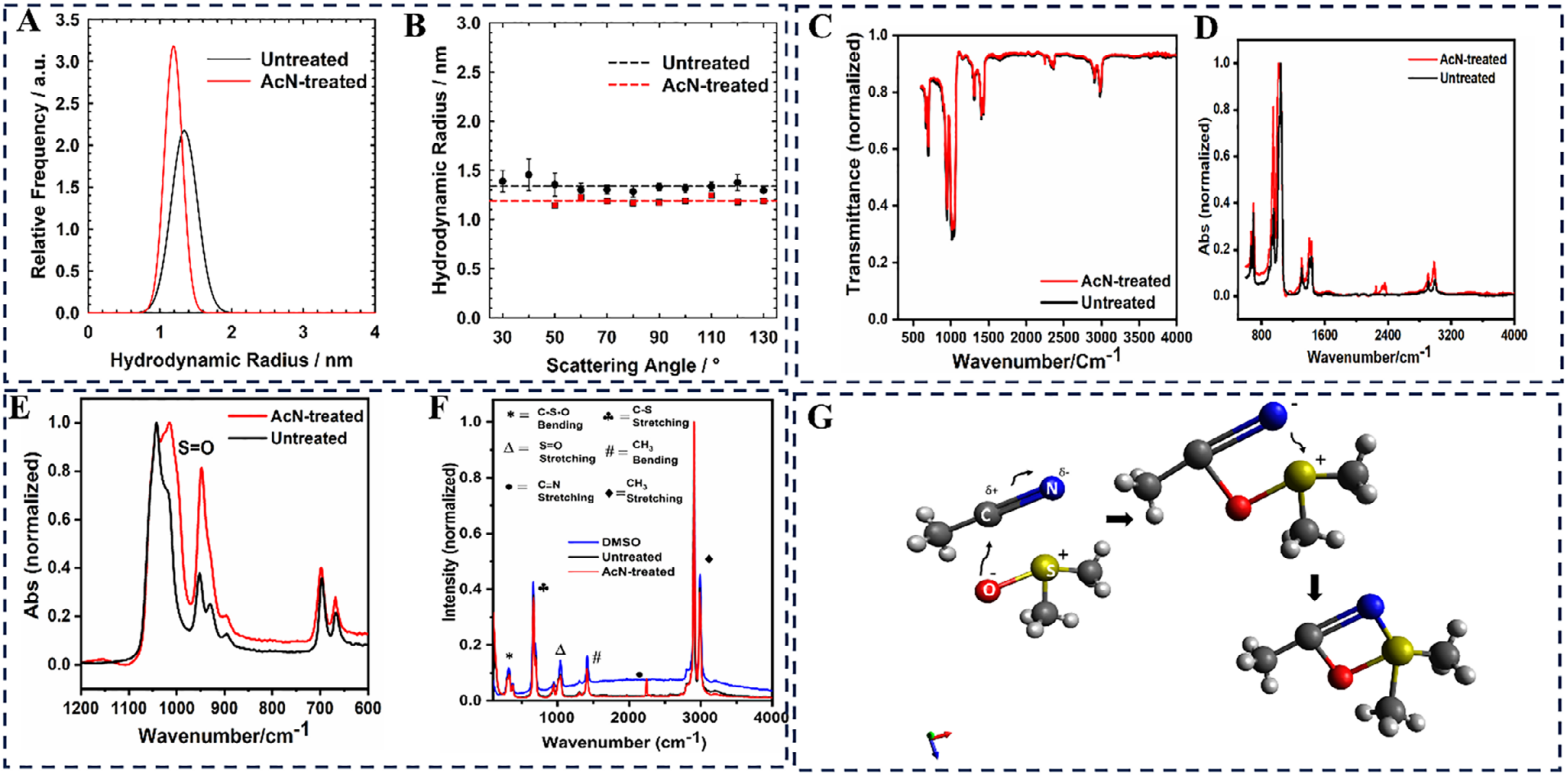
(A, B) represent the dynamic light scattering measurements for the untreated and the AcN‐treated, (C, D) represent the FTIR transmittance and absorbance spectra of the untreated and AcN‐treated, respectively, (E) represents the SIFS with the addition of AcN, (F) represents the Raman spectra of the untreated and AcN‐treated, respectively, (G) represents the reaction mechanism between the DMSO and AcN.

To understand the role of AcN in more detail, different characterizations were conducted. Figure 3A,B shows dynamic light scattering (DLS) measurements providing information about the average colloid size in a liquid. We deduce that the precursor with 10 vol% AcN results in a small but noticeable reduction of the hydrodynamic radius (R_h_) of the perovskite colloids by ∼11% from *R*_*h*, untreated_ = (1.34 ± 0.05 *nm*) *to* *R*_*h*,AcN − treated_ = (1.19 ± 0.04 nm).

This reduction is consistent with a weakened interaction strength between CsPbI_2_Br and DMSO. In other words, in the presence of AcN, DMSO coordinates slightly less to Pb^2+^ [20, 21, 22]. In a suitable solvent, such as DMSO, the perovskite solvates readily, and the solvation shell is large. The presence of solvents that coordinate only weakly, as is the case with AcN, leads to the ions clustering together more strongly to avoid unfavorable interactions with AcN, effectively leading to a smaller solvation shell and therefore a reduced hydrodynamic radius. This reduction can be interpreted by the Stokes–Einstein equation (Equation 1), where the reduced R_h_ corresponds to the faster diffusion of smaller clusters in the presence of AcN.

(1) $$R_{h}=\frac{k_{B}T}{6\pi \eta D}$$

where R_h_ is the hydrodynamic radius, *k_B_* is the Boltzmann constant, η is the viscosity of the continuous solvent in which the particle is dispersed, T is the temperature, and D is the diffusion coefficient.

While an even stronger effect of AcN on *R_h_* might be anticipated, it should be considered that (1) the AcN concentration is still relatively low and that (2) the different interaction gets stronger between Pb^2+^ and DMSO and AcN, respectively, might lead to a concentration gradient of the two solvents, with DMSO being located preferably in the vicinity of the perovskite clusters, whereas AcN would then rather diffuse into the continuous bulk phase. This is problematic if the actual scatterer (e.g., the particle) is not spherical but simply diffuses in the same way as the theoretical sphere. Performing angle‐dependent experiments is therefore advantageous because a non‐spherical particle will produce a different scattering signal if the angle is changed. However, in our case, Figure 3B gives a strong indication of the spherical nature of the CsPbI_2_Br clusters. Within measurement uncertainty, the hydrodynamic radius remains constant for measurements between θ = 30° and 130°. The presence of AcN does not reveal any qualitative deviations from this trend. The interaction between DMSO and AcN is further investigated with Fourier‐transform infrared spectroscopy (FTIR) in Figure 3C,D. Figure 3C shows that there is a slight quenching in the sulfoxide group (S═O) of DMSO. This phenomenon is further supported by the FTIR absorbance spectra in Figure 3D.

As reported earlier by Fawcett et al., the behavioral change of DMSO when mixed with high Gutmann acceptor number solvent is an interesting phenomenon and can be attributed to the Kirkwood‐Bauer‐Magat relation (Equation 2), which helps to not only estimate dipole moments of molecules from dielectric values but also connects molecular microscopic interactions with macroscopic dielectric nature [23, 24, 25, 26, 27, 28, 29].

(2) $$\frac{\left(\varepsilon -1\right)\left(2\varepsilon +1\right)}{\varepsilon }*\frac{M}{\rho }=\frac{\left(9k_{B}T\right)}{\left(4\pi NA\right)}*\frac{\left(\alpha +\mu^{2}g\right)}{3k_{B}T}$$

where ε is the dielectric constant, M is the molar mass, ρ is the density, NA is the Avogadro number, k_B_ is the Boltzmann constant, T is the absolute temperature, α is the molecular polarizability, µ is the dipole moment, and g is the Kirkwood correlation factor. Equation (2) is directly relevant in this case, since the addition of AcN reduces DMSO's self‐aggregation property, hence alters the Kirkwood correlation factor (g), which also expresses the dipole orientation correlations. Additionally, with the alteration of µ and g upon addition of AcN can directly alter the dielectric constant (ε) of pure DMSO. Many groups have shown that, with higher concentration or under specific conditions, DMSO tends to form dipole‐dipole clusters due to dipole–dipole interactions. This may be due to the interaction of the O of DMSO with the H of impurities or the environment. This leads to the self‐aggregation of DMSO molecules, leading to degradation [30, 31]. In this work, while being mixed with AcN, DMSO shows a “solvent‐induced frequency shift” (SIFS) [32, 33, 34] in Figure 3E, i.e., compared to the untreated, for the AcN‐treated sample's FTIR absorbance spectra, the shoulder not only reduced but there is also a spectral shift is observed. This is primarily due to the change in the vibrational environment of S═O of DMSO upon addition of AcN. The strong peak is not observed in the presence of AcN due to the weaker interaction between DMSO anfd AcN, which is in line with the DLS results. One of the primary possibilities of this shift is the formation of the antiparallel dipolar conformation as shown in Figure 3D. As reported earlier, the S═O bond in the DMSO molecule remains as a resonance hybrid of a semipolar double bond with a pπ‐dπ interaction where the oxygen atom is electronegative compared to the sulfur atom.

(3) $${\left(H_{3}C\right)}_{2}S^{+}-\;O^{-}↔{\left(H_{3}C\right)}_{2}S=O$$

As shown in Figure 3E, when the electronegative oxygen atom interacts with the carbon atom (partially charged) of AcN due to the previously mentioned antiparallel dipolar conformation, it helps to polarize the S═O bond of DMSO and accelerate the SIFS. Further, the FTIR transmittance spectra of DMSO, untreated and AcN‐treated perovskite ink can be found in Figure S7. This observation corroborates well with the Raman spectroscopy for the untreated and AcN‐treated as shown in Figure 3F. In pure DMSO, all perovskite precursors are dissolved by pairing ions with S+ and O^−^ of DMSO. However, when AcN is added, it weakly interacts with DMSO due to its low dielectric constant and depolarizes the system due to the reduced dipole moment, due to stabilized delocalization from the AcN‐DMSO solvent system [35, 36]. This delocalization form of AcN‐DMSO attracts more ions to pair together and initiate the crystallization, followed by the appearance of highly polarized and stabilized Raman peaks for the AcN‐DMSO‐perovskite salt mixture (also see Figure S13 for additional nuclear magnetic resonance data). When AcN is added, rotational vibrational modes of DMSO molecules are enhanced (Raman spectra of the AcN‐DMSO mixture), but it is damped and compensated with pairing with high‐order ionic species around it (nuclei or seeding of crystals). These nuclei are directionally grown crystals and highly ordered species due to ionic solvent complex formation. These coordinated nuclei are defect‐free, followed by controlled crystallization after annealing. Hence, with the co‐solvent strategy, the perovskite solution (AcN‐treated) gives better performance film than a pure DMSO‐based perovskite solution (untreated). We further assume that, during this interaction, DMSO and AcN partially create an intermediate complex (in Figure 3G), which hinders DMSO from interacting solely with the Pb^2+^ of the perovskite and alters DMSO's coordination by supporting [DMSO‐AcN] complex formation. This way, the removal of DMSO becomes easier during the crystallization stage of the perovskite absorber layer, followed by obtaining a higher‐quality perovskite absorber layer.

**FIGURE 4:**
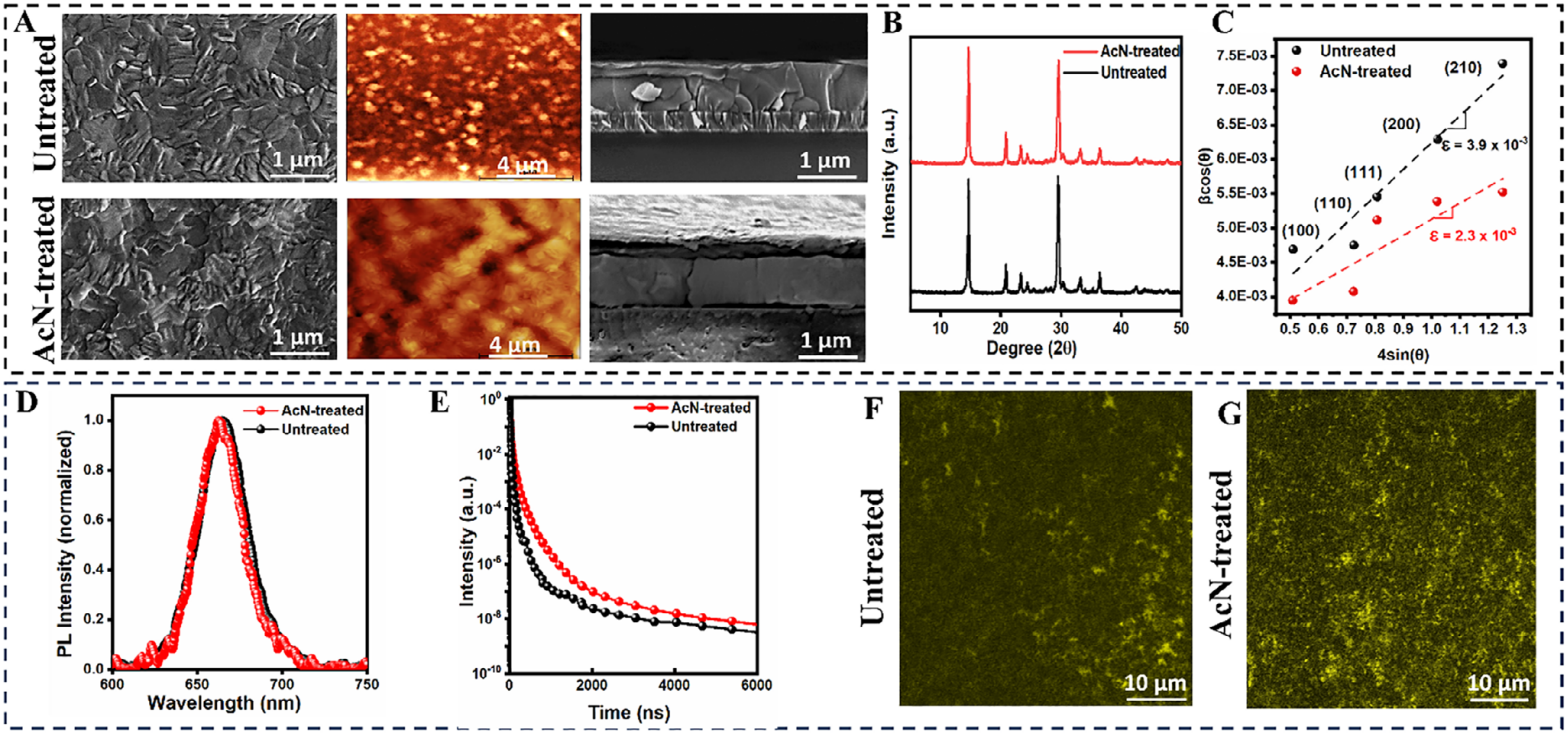
(A) represents the SEM top‐view, AFM, and SEM cross‐section of the untreated and the AcN‐treated respectively, (B, C) represent XRD and microstrain calculations for the untreated and AcN‐treated films; (D) represents the steady‐state PL spectra; (E) represents the TRPL measurements of the AcN‐treated and untreated films, respectively; (F, G) represent the cathodoluminescence images for the untreated and AcN‐treated film respectively.

To further investigate the effect of AcfN on the thin film morphology, scanning electron microscope (SEM) top‐view and cross‐section measurements are performed for the untreated and AcN‐treated films. As seen in Figure 4A, the film morphology improved significantly with the addition of AcN with the increment in average grain size from 114.5 nm (untreated) to 121.2 nm (AcN‐treated), as shown in Figure S8. This is further supported by the atomic force microscope (AFM) measurement, where the surface roughness, i.e., the root‐mean‐square value, decreased from 65 ± 0.5 to 51 ± 0.5 nm. This result supports our DLS and FTIR data about obtaining better film quality with the addition of AcN into DMSO. In addition, this follows the trend in current density increase as the charge carrier extraction is improved due to the higher quality of the perovskite absorber by the treatment (as shown in Figure 2B). To further analyze the influence of AcN, X‐ray diffraction (XRD) measurements are performed as shown in Figures 4B and S9. As seen in Figure 4B, the perovskite peak intensity is slightly increased for the AcN‐treated films compared to the untreated films. The comparison of full width at half‐maximum (FWHM) between untreated and AcN‐treated (Figures 4C and S9) from 0.29 to 0.27 reveals differences attributable to variations in average d‐spacing at specific Bragg reflections. To quantitatively assess strain‐induced peak broadening across different diffraction angles (2θ), we employ the Williamson–Hall equation (Equation 4), which is used for microstrain analysis:

(4) $$\beta_{hkl}cos\left(\theta \right)=\left(k\lambda /D\right)+4\varepsilon \sin\left(\theta \right)$$

β_hkl_ is the integral breadth of the XRD peak (full width at half maximum, FWHM), k is the Scherrer constant, λ is the X‐ray wavelength, D is the crystallite size, and ε denotes the microstrain. In this approach, the slope of the linear fit of β_hkl_ cosθ vs. 4sinθ directly corresponds to the microstrain present in the perovskite thin film [37]. As shown in Figure 4C, the extracted microstrain decreases from 3.9 × 10^−3^ to 2.3 × 10^−3^ in the AcN‐treated film, representing a ∼ 40% reduction. This decreasement is indicative of diminished local lattice distortions and enhanced compositional homogeneity, which are attributed to the incorporation of AcN in the perovskite precursor ink [38]. This finding is further corroborated by the full‐width at half‐maximum (FWHM) of both the untreated and the AcN‐treated films (Figure S10). The FWHM of the untreated is 0.29 with a broader peak, whereas for the AcN‐treated film it is 0.27 with a narrower peak, which indicates for the AcN‐treated the perovskite film quality is improved, leading to better crystallinity, which corroborates well with the PV parameters improvement as was previously mentioned in Figure 2B. To further analyze the photoluminescence properties, steady‐state photoluminescence (ss‐PL) and time‐resolved PL (TRPL) were performed as shown in Figure 4D,E. For ss‐PL measurement (Figure 4D), as can be seen, both the untreated and AcN‐treated films have comparable PL‐emission intensities, suggesting a comparable radiative efficiency. However, the AcN‐treated film shows a slightly narrower peak compared to the untreated film, indicating reduced defect states for the AcN‐treated film. To further understand the charge‐carrier dynamics, TRPL measurements were performed.

For both the untreated and AcN‐treated, the TRPL decay curves are fitted with a biexponential decay function:

(5) $$I\left(t\right)=A_{1}\;exp\left(-\frac{t}{\tau_{1}}\right)+A_{2}\;exp\left(-\frac{t}{\tau_{2}}\right)+K$$

The average PL decay times (τavg) are further estimated using the following expression:

(6) $$\tau_{avg}=\frac{A_{1}\tau_{1}^{2}+A_{2}\tau_{2}^{2}}{A_{1}\tau_{1}+A_{2}\tau_{2}}$$

here, A_1_ and A_2_ are the decay amplitudes, and τ_1_ and τ_2_ are the characteristic time constants for the first and second exponential decays, respectively. K is a constant for the baseline offset. Here, during the analysis, τ_1_ and τ_2_ represent the characteristic decay times, which are driven by factors such as trap states or charge extraction. The fast decay component (τ_1_) is attributed to non‐radiative or trap‐assisted recombination, and the slow decay (τ_2_) is attributed to radiative or direct recombination. The untreated film shows a relatively steeper fast decay and lower long‐time tail compared to the AcN‐treated film. This indicates that trap‐assisted recombination dominates for the untreated film. On the contrary, for the AcN‐treated film, both fast decay is reduced, and a relatively higher long‐time tail can be observed, which indicates reduced trap‐assisted recombination (in Figure 4E) followed by an increment in the *V_OC_* for the AcN‐treated device as previously observed in Figure 2B, as well as reduced hysteresis (Figure S2). This is further corroborated by the ultraviolet–visible absorption measurement (Figure S11). Figure 4F,G represents the cathodoluminescence images for untreated and AcN‐treated films. The untreated film (Figure 4F) shows less luminescence upon exposing the film surface to the laser; the AcN‐treated film (Figure 4G) shows much brighter and uniform luminescence, which supports the better film quality obtained by the AcN treatment.

**FIGURE 5:**
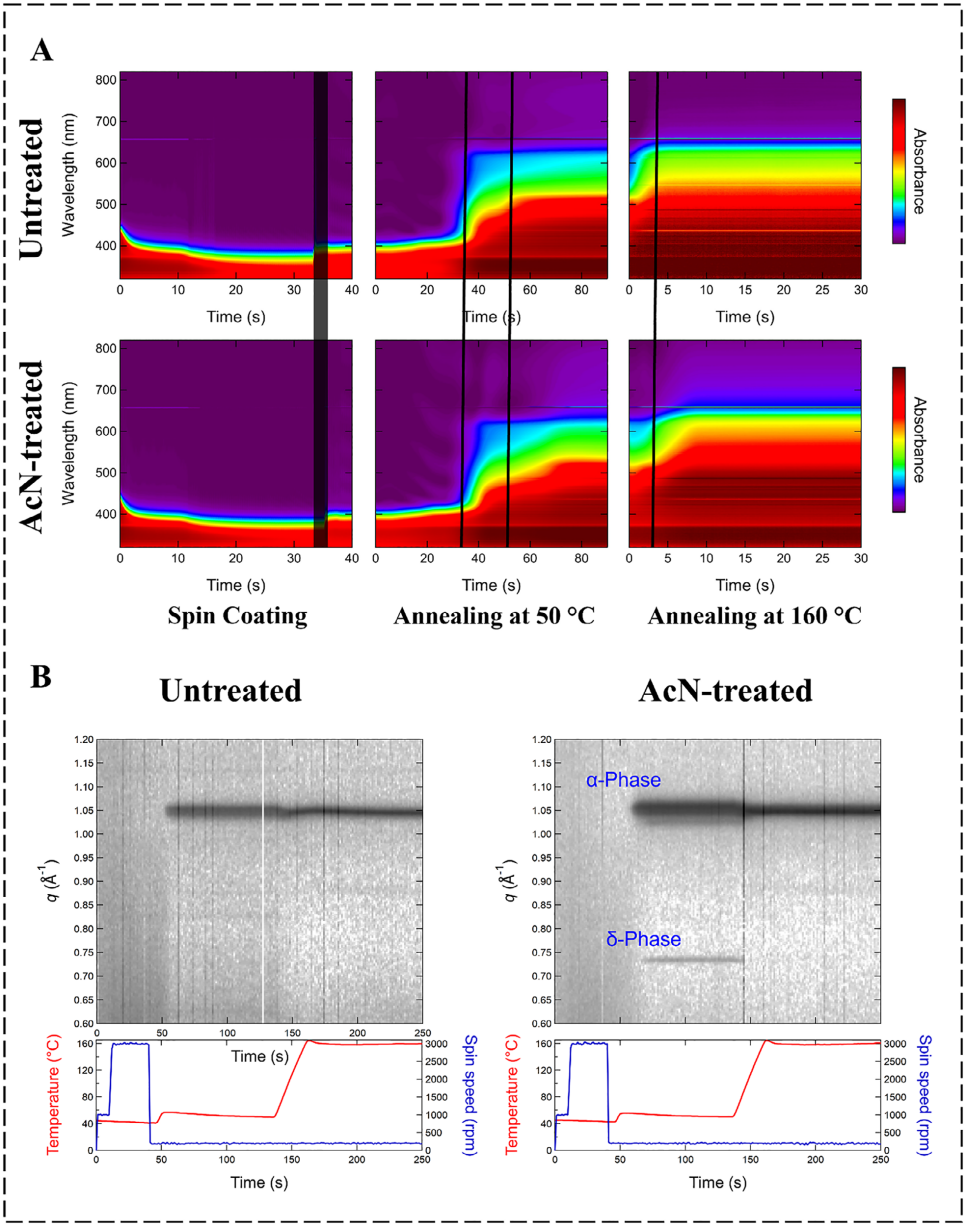
(A) shows heatmaps of the absorbance of the untreated (top row) and AcN‐treated samples (bottom row) measured in situ during spin‐coating and annealing. The vertical lines represent the dropping of the antisolvent, as well as the appearance of absorption features to serve as a guide for the eye. (B) shows the respective evolution of the structural properties of both samples measured with in situ GIWAXS. The spin coating and temperature profiles are depicted at the bottom of each panel.

To understand the improved film quality more deeply, we conducted in situ absorbance and in situ grazing incidence wide‐angle X‐ray scattering (GIWAXS) experiments. Figure 5A shows that in both cases, the film deposition starts with the development of a wet precursor film with an absorbance edge around 450 nm, shifting to about 400 nm during spin‐coating. Dropping of the chlorobenzene and the subsequent low‐temperature annealing step at 50°C induce supersaturation of the solution and subsequent nucleation of a thin film with a red‐shifted absorbance edge at around 500–600 nm. Finally, during high‐temperature annealing, the absorbance edge redshifts again to its final position at about 600 nm. We interpret this evolution of the absorbance edge as the nucleation of small perovskite crystallites and their subsequent growth toward a closed, polycrystalline thin film. Figure 5A shows that this evolution is slightly delayed in the case of the AcN‐treated sample, indicating slowed nucleation and crystal growth, which is usually attributed to more homogeneous nucleation and larger grain growth.

The in situ GIWAXS data shown in Figures 5B and S12 supports this hypothesis. In both cases, a diffraction peak at q ≈ 1.05 Å^−1^ corresponding to the α‐perovskite phase appears during the low‐temperature annealing step right after the end of the spin‐coating process. Again, the appearance of this peak, associated with the formation of the perovskite crystallites, is slightly delayed for the AcN‐treated sample. This is further corroborated by the GIWAXS intensity vs time plot as shown in Figure S12. Additionally, there is an intermediate peak appearing at q ≈ 0.73 Å^−1^ in the case of the AcN‐treated sample that is not present in the untreated. We attribute this peak to the metastable delta‐phase perovskite, which, after annealing processes, could completely convert to the pure α‐perovskite phase. This could also be related to the [DMSO‐AcN] complex formation.

We speculate that AcN in the mixed solvent partially reacts with DMSO due to its electron‐accepting capacity [39, 40, 41]. This condition leads to the uncoordinated DMSO reacting partially with the AcN, leading to the formation of the [DMSO‐AcN] coordination. This coordination complex formation leads to the reduction of the uncoordinated DMSO from the solution, leading to a perovskite layer obtained is of higher quality, which is reflected in the improved device performance (PCE) increment from 12.7% to 14%.

**FIGURE 6:**
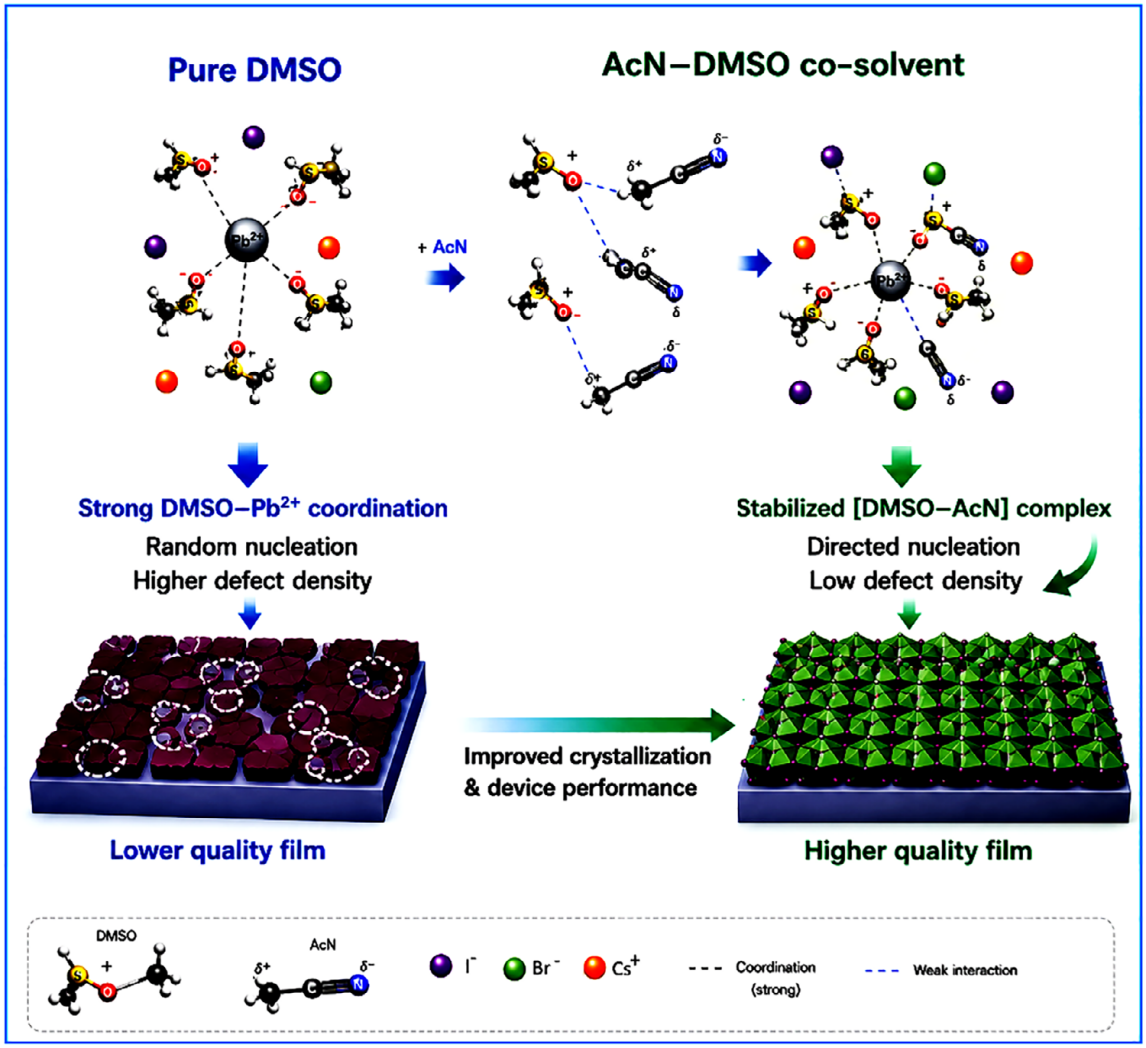
Schematic illustration of the proposed AcN–DMSO co‐solvent mechanism for perovskite crystallization. The AcN‐induced modification of DMSO coordination promotes ion pairing and directional nucleation, facilitates DMSO removal during annealing, and results in controlledf crystallization and a high‐quality, low‐defect perovskite film.

Based on the experimental phenomena and analysis so far, Figure 6 schematically illustrates the proposed role of the AcN co‐solvent during perovskite crystallization. In pure DMSO, the perovskite precursors are strongly solvated through DMSO coordination, resulting in a highly dissolved precursor state and delayed nucleation. Upon introducing AcN, weak AcN–DMSO interactions partially modify the DMSO coordination environment, promoting the formation of a transient DMSO–AcN solvent complex. This interaction reduces the effective solvation strength of DMSO toward Pb^2+^, facilitating ion pairing, directional nucleation, and more ordered crystal growth. During annealing, the intermediate solvent complex enables easier DMSO removal, leading to controlled crystallization, reduced defect density, and the formation of a dense, high‐quality perovskite film with improved optoelectronic properties. Moreover, to compare our work with other co‐solvent engineering strategies for CsPbI_2_Br composition, Table S1 is provided [42, 43, 44, 45, 46].

## 3 Conclusion

In conclusion, we demonstrate a solvent engineering strategy to further improve the efficiency and stability of all‐inorganic CsPbI_2_Br perovskite solar cells. We have observed that the addition of AcN along with DMSO during the perovskite solution preparation can slow down the crystallization. We speculate that the addition of AcN in DMSO solvent reduces the uncoordinated DMSO and tends to create a [DMSO‐AcN] complex in addition to the existing [DMSO‐Pb] complex, resulting in better perovskite absorber layer formation, which is evident especially from the DLS and FTIR reports. Additionally, this solvent engineering strategy results in better device performance, giving a significant boost in all device parameters, leading to an efficiency boost from 12.7% (untreated) to 14.0% (AcN‐treated).

## Supporting information




**Supporting File**: advs77395‐sup‐0001‐SuppMat.docx.

## Data Availability

The data that support the findings of this study are available from the corresponding author upon reasonable request.
