# Supplementary material for: Effective Solvent‐Engineered All‐Inorganic CsPbI2Br Perovskite Solar Cells
Source: Adv Sci (Weinh). 2026 Aug 27;13(63):e77395. doi: 10.1002/advs.77395 (PMC13521201; doi:10.1002/advs.77395)
Supplement: Supplementary file 1 [file ADVS-13-e77395-s001.docx]

Supporting Information

**Effective Solvent-Engineered All-Inorganic CsPbI_2_Br Perovskite Solar Cells**

Rajarshi Roy**^1^**^‡^, Mahdi Malekshahi Byranvand**^1,2^***^‡^, Tim Kodalle**^3,4^**, Maximilian Krappel**^5^**, Laxman Gouda**^6^**, Stephan Boehringer**^1^**, Chittaranjan Das**^1^**, Mayank Kedia**^1,2^**, Carolin M. Sutter-Fella**^3,4^**, Michael Saliba**^1,2^***

^1^Institute for Photovoltaics (ipv), Research Center SCoPE, and Integrated Quantum Science and Technology Center (IQST), University of Stuttgart, Pfaffenwaldring 47, 70569 Stuttgart, Germany^2^Helmholtz Young Investigator Group FRONTRUNNER, IMD-3 Photovoltaics, Forschungszentrum Jülich, 52425 Jülich, Germany

^3^Molecular Foundry, Lawrence Berkeley National Laboratory, Berkeley, CA, USA

^4^Advanced Light Source, Lawrence Berkeley National Laboratory, Berkeley, CA, USA

^5^Institute of Physical Chemistry, University of Stuttgart, 70569, Germany

^6^CeNSE Department, Indian Institute of Science, 560012, Bangalore, India

Email : [mahdi.malekshahi@ipv.uni-stuttgart.de](mailto:mahdi.malekshahi@ipv.uni-stuttgart.de), michael.saliba@ipv.uni-stuttgart.de

**^‡^** These authors contributed equally to this work.

**Experimental Section**

**Device fabrication**

The CsPbI_2_Br perovskite solar cells (PSCs) were fabricated in n-i-p solar cell planar architecture:

Glass substrate, indium tin oxide (ITO)/SnO_2_ nanoparticle-electron transport layer (ETL)/CsPbI_2_Br perovskite absorber (with DMSO solvent)/ Poly(3-hexylthiophene) (P3HT) hole transport layer (HTL)/gold back electrode for the control devices and indium tin oxide (ITO)/nanoparticle-based SnO_2_ electron transport layer (ETL)/ CsPbI_2_Br perovskite absorber absorber (with DMSO:AcN co-solvent)/ Poly(3-hexylthiophene) (P3HT) hole transport layer (HTL)/gold back electrode for the target devices.

For the device fabrication, Indium tin oxide (ITO) substrates with sheet resistance 15 Ω sq−1, (Luminescence Technology) were cleaned thoroughly with deionized (DI) water, acetone (Sigma Aldrich), and isopropyl alcohol (Sigma-Aldrich) in an ultrasonic bath for 15 min for each step followed by UV-ozone treatment for 30 min. For the electron transport layer (ETL) deposition, a very thin layer of 2.04 wt. % colloidal SnO_2_ nanoparticles (Alfa Aesar, 15 wt. % aqueous solution) solution was spin-coated on the thoroughly washed ITO substrates with 4000 rpm for 30 s followed by annealing at 150 °C for 30 min. For the perovskite absorbing layer deposition, we have deposited CsPbI_2_Br (1.3M) perovskite solution following a fixed stoichiometry of PbBr_2_ (TCI Chemicals), PbI_2_ (TCI Chemicals), and CsI (Alfa Aesar) in the DMSO solvent (Sigma Aldrich) over the ETL layer. The perovskite solution was spin-coated for 30 s at 3000 rpm followed by the dynamic deposition of chlorobenzene as an antisolvent for the control substrates. Otherwise, different ratios of DMSO: acetonitrile (AcN) (Alfa Aesar) (900:100/850:150/800:200) co-solvent were used to dissolve the perovskite precursors to finally obtain the perovskite solution (target) followed by the similar spin-coating recipe as mentioned earlier. For the target, the optimized ratio (900:100) of DMSO:AcN co-solvent had been used. For both cases, we have annealed the substrates for 1.5 min at 50 °C followed by 160 °C for 10 min. For the HTL deposition, P3HT solution in CB was prepared and spin coated at 2500 rpm for 25 s followed by annealing at 200 °C for 5 min. Finally, 80 nm thick gold (Au) electrode was deposited by thermal evaporation with a shadow mask with cell area of 0.16 cm^2^.

**Characterization**

The solar cells were characterized by a 21-channel LED Solar Simulator (Wavelabs Solar Metrology Systems) with an AM1.5G spectrum (100 mW/cm^2^). The *J-V* measurements were performed with a constant scan rate of 10 mV/s (Keithley 2400 source measurement unit). The stabilized power output (SPO) of different solar cells was measured by applying a constant maximum power point voltage over a certain time period. The perovskite layer crystallinity was examined by an X-Ray diffraction (XRD, Panlytical) machine. The XRD patterns were measured on an X′′Pert Pro (PANanalytical) in Bragg–Brentano geometry using Cu Kα1 radiation (*λ* = 1.5406 Å) and scanning from 2° to 60° (2*θ*) with a step interval of 0.0167°.The UV-Vis absorbance spectra were measured by using the PerkinElmer LAMDA 1050. The scanning electron microscope (SEM) from HITACHI was used to characterize the different morphologies of the perovskite films with an accelerating voltage of 3 kV. Atomic force microscopy (AFM) measurement was performed in a closed nitrogen atmosphere. The topology was acquired in frequency modulation (AM).

The photoluminescence setup consists of a 405 nm picosecond diode Laser (PicoQuant LDH-P-C 405), capable of up to 80 MHz repetition rate and a pulse width of 44 ps. The average power is up to 4.6 mW and a peak power of 660mW. The sample is positioned by a motorized X,Y,Z-Stage under a parabolic collection mirror into the focal point, this allows a high numerical aperture and absence of chromatic aberrations. The laser is focused through a hole in the collection mirror onto the sample surface achieving a focal spot diameter of <50um. This arrangement allows the separating of the excitation beam path from the signal beam path. The collected light then enters a Czerny-turner equipped with a PicoQuant PMA Hybrid 50 (380-890nm) single photon counter, a motorized filter wheel is placed in front of the entrance slit with notch filters for 405nm and bandpass filters (suppression of higher order reflections on the grating).

Steady-state as well as time resolved measurements are taken with this detector. By scanning the grating of the Czerny turner, a spectrum can be acquired. For time resolved measurements the Czerny turner is moved to the wavelength of interest and the decay curve is reconstructed by time correlated single photon counting (TCSPC) (PicoQuant TimeHarp 260 PICO Single).

Dynamic light scattering (DLS) measurement was performed for both control and target devices.

**Experimental**: Dynamic light scattering (DLS) experiments were recorded with the LS Spectrometer by LS Instruments, Switzerland. The spectrometer is equipped with a diode-pumped laser operating at a wavelength of $\lambda$ = 561 nm (Cobolt, Sweden). Decalin is used as index matching fluid, with the temperature of the decaling bath being regulated via a thermostat (Julabo, Germany) to adjust a measurement temperature of *T* = (25.0 $\pm$ 0.1) °C. Samples were filled into borosilicate test tubes (Fisher Scientific, USA). The incident laser intensity was set to maximum ($I_{0,max}\approx$ 81 mW) in order to obtain the highest possible detector count rates. Measurement protocol was three repetitions with 150 s each for all targeted scattering angles, ranging from $\theta$ = 30 – 130° in steps of 10°. All measurements were run in 2D pseudo cross correlation mode, incorporating a second APD detector in order to mitigate the effect of after-pulsing originating from detector noise.

**Theory**: Dynamic light scattering (DLS), also known as photon correlation spectroscopy (PCS) or quasi-elastic light scattering (QELS), can be used to investigate the diffusion behavior of particles or polymers in solution, allowing for the determination of the hydrodynamic radius. Detailed information on development, theory and applications can be found in a review article by Stetefeld et al *[doi.org/10.1007/s12551-016-0218-6]*. For this study, DLS measurements were performed as a function of the scattering angle $\theta$, which in turn provides different wave vectors $q$, with

$$q=\frac{4\pi n}{\lambda}\cdot\sin\left( \frac{\theta}{2} \right),$$

where $\lambda$ is the laser wavelength and $n$ is the solvent refractive index. The obtained intensity-time autocorrelation function as a function of wave vector and lag time $\tau$ is denoted as

$$g_{2}\left( q;\tau\right)=\frac{<I\left( t \right)I\left( t+\tau\right)>}{<I\left( t \right)>^{2}}.$$

It can be related to the field-time correlation function $g_{1}(q;\tau)$ via the Siegert relation

$$g_{2}\left( q;\tau\right)=1+\beta\left[ g_{1}\left( q;\tau\right) \right]^{2},$$

where $\beta$ is an instrument- and measurement-specific correction factor. For monodisperse size distributions, it can be assumed that

$$\left| g_{1}\left( q;\tau\right) \right|=exp(-\Gamma\tau)$$

with the decay rate $\Gamma$, which can be further used to calculate the (translational) diffusion coefficient $D$ via

$$\Gamma=Dq^{2}.$$

The Cumulant method developed by Koppel can be used to analyze the intensity-time correlation function in order to determine the (Gaussian) distribution around the average particle size [*doi.org/10.1063/1.1678153*]. Since DLS investigates the particle diffusion in a solvent, the obtained particle size is the hydrodynamic radius $R_{h}$, which can be determined from the Stokes-Einstein equation as

$$R_{h}=\frac{k_{B}T}{6\pi\eta D},$$

where $k_{B}$ is the Boltzmann constant and $\eta$ is the viscosity of the continuous solvent in which the particle is dispersed [*doi.org/10.1021/je8001909*].

FTIR measurements were conducted with Bruker alpha II spectrometer and data analysed using OPUS TOUCH software.

The in situ GIWAXS measurements during spin coating and annealing were carried out in a custom-made spin-coater at beamline 12.3.2 at the Advanced Light Source (ALS), Lawrence Berkeley National Laboratory. The incoming X-ray beam was set to an angle of 2° using a beam energy of 10 keV. The spot size of the beam was approximately 5 um by 115 um. A DECTRIS Pilatus1 M detector at an angle of 35° to the sample plane and a sample to detector distance of ∼ 190 mm were used. The samples were deposited via spin-coating and annealing on a glass/ITO substrate in an N_2_-flushed chamber while continuously collecting diffraction data with a time resolution of about 1.4 s. Data analysis was carried out using custom software deployed at the beamline (“XRDSol”) as well as a Python script based on the packages numpy [DOI:[*10.1038/s41586-020-2649-2*](https://doi.org/10.1038/s41586-020-2649-2)], pandas [DOI:*10.5281/zenodo.3509134*], matplotlib [DOI:*10.1109/MCSE.2007.55*], and scipy [DOI:*10.1038/s41592-019-0686-2*].

The in situ GIWAXS measurements during spin coating and annealing were carried out in a custom-made spin-coater at beamline 12.3.2 at the Advanced Light Source (ALS), Lawrence Berkeley National Laboratory. The incoming X-ray beam was set to an angle of 2° using a beam energy of 10 keV. The spot size of the beam was approximately 5 um by 115 um. A DECTRIS Pilatus1 M detector at an angle of 35° to the sample plane and a sample to detector distance of ∼ 190 mm were used. The samples were deposited via spin-coating and annealing on a glass/ITO substrate in an N_2_-flushed chamber while continuously collecting diffraction data with a time resolution of about 1.4 s. Data analysis was carried out using custom software deployed at the beamline (“XRDSol”) as well as a Python script based on the packages numpy [DOI:[*10.1038/s41586-020-2649-2*](https://doi.org/10.1038/s41586-020-2649-2)], pandas [DOI:*10.5281/zenodo.3509134*], matplotlib [DOI:*10.1109/MCSE.2007.55*], and scipy [DOI:*10.1038/s41592-019-0686-2*].

**Supporting figures**

Fig S1. Device parameters (A) *J*_SC_, (B) *V_OC_*, (C) *FF*, (D) *PCE* extracted from *J-V* measurements from the series of control and varied ratios of AcN:DMSO co-solvated devices.

**Fig S2.** Represents hysteresis index for untreated and AcN-treated device with 50 mV/s scan rate.

**Fig. S3** (A-D) represent device statistics of the fresh and aged untreated devices for *J*_SC_, *V_OC_*, *FF*, *PCE* at 200 °C for 240 min (n = 7).

**Fig. S4** (A-D) represent device statistics of the fresh and aged AcN-treated devices for *J*_SC_, *V_OC_*, *FF*, *PCE* at 200 °C for 240 min (n = 7).

**Fig. S5** (A-D) represent device statistics of the fresh and aged untreated devices for *J*_SC_, *V_OC_*, *FF*, *PCE* at RH ~ 60 % for 180 min (n = 7).

**Fig. S6** (A-D) represent device statistics of the fresh and aged AcN-treated devices for *J*_SC_, *V_OC_*, *FF*, *PCE* at RH ~ 60 % for 180 min (n = 7).

**Fig S7.** FTIR transmittance spectra of DMSO solvent, untreated and AcN-treated films.

**Fig S8.** Mean grain size of the perovskite films with and without the AcN addition.

**Fig S9.** XRD measurements of perovskite films with and without the addition of AcN.

**Fig S10.** Full-Width-Half-Maxima (FWHM) of the untreated and AcN-treated films.

**Fig S11.** UV-vis absorbance measurements of the untreated and different ratios of the AcN : DMSO films over the 500 nm-700 nm wavelength range.

**Fig S12.** (A) represents the absorbance in situ during spin coating and annealing for untreated (solid lines) and AcN-treated (dashed lines), (B) represents the nucleation during annealing for untreated and AcN-treated, (C) represents the final absorption edge for the untreated AcN-treated films respectively.

**Fig S13.** (A) ^1^H NMR and (B) 13C NMR spectra of the perovskite films with and without the AcN addition.

**Table S1** Literature comparison of co-solvent engineering strategy for CsPbI_2_Br composition

| Co-solvent Engineering | Perovskite | Major findings | Year | Best PCE % | References |
| --- | --- | --- | --- | --- | --- |
| DMSO:DMF | CsPbI_2_Br | DMSO-controlled volume tuning leads to smoother, thicker films | 2017 | 10.8 | [43] |
| DMSO:DMF +Aminothiourea | CsPbI_2_Br | Precursor solubility improved | 2022 | 13.36 | [44] |
| DMSO:GBL | CsPbI_2_Br | Controlled crystallization | 2023 | 14 | [45] |
| DMSO:AcN | CsPbI_2_Br | Controlled crystallization, improved film morphology, reduced trap assisted recombination | 2026 | 14 | **This Work** |
| DMSO:NMP | CsPbI_2_Br | Slower crystallization | 2022 | 17 | [46] |
| DMSO only | CsPbI_2_Br | Lewis base adduct assisted crystallization | 2018 | 13.54 | [47] |
